# Distress and Psychosocial Needs in Patients Accessing a Cancer Day Surgery Division: Implications for Clinical Decision Making

**DOI:** 10.3389/fpsyg.2016.02040

**Published:** 2016-12-27

**Authors:** Paola Arnaboldi, Silvia Riva, Valeria Vadilonga, Liliana Tadini, Giorgio Magon, Gabriella Pravettoni

**Affiliations:** ^1^Applied Research Division for Cognitive and Psychological Science, European Institute of OncologyMilan, Italy; ^2^Department of Oncology and Hemato-oncology, Faculty of Medicine, University of MilanMilan, Italy; ^3^Day Surgery Division, European Institute of OncologyMilan, Italy; ^4^Service for Nursing, Technical and Rehabilitation Staff, European Institute of OncologyMilan, Italy

**Keywords:** distress, distress thermometer, decision making, needs assessment, psychological assessment

## Abstract

**Introduction:** The Distress Thermometer (DT) was built and validated for screening cancer patients for distress, as suggested by the National Comprehensive Cancer Network. The current work was designed to measure the rates of distress in a sample of patients being hospitalized in a multidisciplinary outpatient surgery clinic. Objective: To measure the rates of distress in a sample of patients referring to a multidisciplinary day surgery division in a comprehensive cancer center based in Northern Italy.

**Methods**: A total of 177 patients were asked to fill in the (DT) before surgery.

**Results**: Out of 177 patients, 154 (87%) patients completed the DT. While 13% of the patients indicated a total absence of distress, more than half of the sample declared a moderate or high distress. A total of 55% of patients presented at least three difficulties in the Problem List Checklist. Distress was not correlated with age or other medical and clinical variables. Number of emotional problems was the best predictor of distress at admission (β = 0.655, *p* = 0.000).

**Conclusion**: Screening for distress in a day surgery multidisciplinary oncology division is feasible and a relevant percentage of patients can be identified as clinically distressed. Outcomes also highlight the impact of age and precise physical and psycho-social signs as prognostic indicators of clinically significant distress. Measurement of distress and associated problems list represent the preliminary endpoint toward adequate recommendations that contribute to taking care of distress in cancer patients in cost-effective clinical setting.

## Introduction

Recent evidence from the literature underlines a prevalence of psychological distress among cancer patients which ranges between 30 and 40% (Mitchell et al., 2011). Such distress in 31.9% of cases meets the criteria for DSM V mental disorders such as any anxiety disorder, any adjustment disorder, any mood disorder, or somatoform disorder and needs specialist referral to be properly addressed and treated (Mehnert et al., 2014). The prevalence of distress has been shown to vary across major tumor entities and oncology settings, being higher among breast, head and neck, and colon-rectum cancer patients (Zabora et al., 2001). Stress needs to be managed accurately in order to provide efficient patient treatment and appropriate clinical decision making. Different consequences related to stress that may hinder the clinical decision making processes has been identified in the literature. Such consequences include: poor comprehension of medical conditions (Kentish-Barnes et al., 2009), a lower propensity for cognitive reflection, which has been shown to be implicated in reasoning and decision-making (Baldi et al., 2013), the perceptions of insufficiently supportive staff, and poor communication with clinicians (Corrigan et al., 2007).

In 2012 the Distress Management Guidelines Panel within the National Comprehensive Cancer Network in the United States devised the distress thermometer (DT) and the Problem List (PL) that, over the past decade, has been applied in several contexts with evidence of its validity as a screening instrument [National Comprehensive Cancer Network (NCCN), 2013]. The first nationwide validation study in Italy was carried out by Grassi et al. (2013) and confirmed the validity of the DT as a screening tool and its sensitivity and specificity for the detection of patients with emotional distress.

Nowadays, despite the growing recognition of the impact of distress on quality of life, adherence to cancer treatment and on compliance, the mandate of the NCCN regarding evidence-based policy for routine distress screening and proper evaluation and referral continue to lack (Lazenby et al., 2015).

This is above all true for such settings such as day surgery facilities for cancer patients. Such facilities have been implemented in recent decades as a consequence of cost-effective considerations: ongoing cost pressures warrant an examination of cases in which expensive inpatient care might not be the most cost-effective option. Thus, from a cost effectiveness perspective, day surgeries can be preferable to inpatient surgeries (when a bed is allocated and dedicated to a patient to recover after the operation) in situations where patient clinical outcomes are not compromised. The term “day surgery” refers to the clinical, organizational, and administrative possibility to perform a safe surgical procedure in a limited to 1-day inpatient activity. Day surgery differs from outpatients activities because in the latter only local anesthesia is performed. It is important to note, in this regard, that studies in the literature do not consider psychological distress among the clinical outcomes that are not to be compromised (Rahal et al., 2014) even though psychological distress has been demonstrated to have an impact on quality of life and medical costs (Carlson and Bultz, 2003).

One of the most common oncology specialties in which day surgery is performed is breast cancer. In a systematic review of day surgery for breast cancer (Marla and Stallard, 2009) whose aim was to establish the benefits and disadvantages of day surgery for breast cancer, the psychosocial outcomes were very poorly addressed: only one study (Margolese and Lasry, 2000) considered quality of life as measured through a validated questionnaire and this showed better psychological and emotional adjustment in the day surgery group even though 22 of the 55 day patients (40%) would have liked to have spent one night in the hospital rather than going home on the same day, while only 4 of the 35 inpatients (12%) would have liked the procedure to be done as a day patient rather than staying in. These results were replicated in the study by Athwal et al. (2015) where patients’ anxiety and emotional well-being in a day surgery setting was independent of type of surgery and where the vast majority of patients found the overnight stay in hospital following surgery useful.

The main purpose of this study was to fill in the lack of psychological distress screening data in oncology day surgery facilities by administering the Italian version of the DT and of the related PL to patients admitted to surgery in a Comprehensive Cancer Center Day Surgery Division and to study the characteristics of distress prevalence in this specific setting, laying down the basis for implementing proper policy of addressing the issue.

## Materials and Methods

### Study Procedure

Adhering to National and International guidelines, since 2013 each patient admitted to the European Institute of Oncology (IEO) either in an inpatient and outpatient setting has been asked to fill in the DT and the PL, mainly in a self-administered manner or with help from the case manager and primary nurses.

Since May 2010, the IEO has been developing a Day Surgery Division but until now, no distress management policy has been applied to patients admitted to it for treatment. Usually, nurses and physicians refer patients who show greater psychological distress to the Psychology Division for further assessment and examination without administering a screening tool.

In 2015, we decided to integrate a full distress screening management policy and we decided to consider a 1-week sample, screening for psychological distress in every patient accessing the Day Surgery Division between March 23 and March 30, 2015.

When entering the division each patient was asked by their primary nurse to fill in the DT and the PL. Every day of the study week the head nurse gave questionnaires to the psychology division for data management and analysis. Prior to the screening week, two clinical psychologists met with day surgery division nurses to teach them about the purposes and usefulness of psychological distress screening in two educational sessions of 45 min each.

Training sessions were conducted during the nursing delivery on an interactive basis and the screening tool was introduced to nurses by means of cases from psycho-social clinical practice as described in the literature (Grassi et al., 2011).

### Questionnaires

The DT is a visual-analog instrument whereby patients are asked to rate their level of distress in the past week on a scale from 0 (no distress) to 10 (extreme distress). The PL consists of a list of 34 problems grouped into 5 categories, comprising practical problems, emotional problems, spiritual/religious concerns, and physical problems. The PL is rated in a dichotomic yes/no format (Grassi et al., 2013).

The Italian validation study of the instrument (Grassi et al., 2013) revealed that the DT cut-off scores ≥5 had optimal sensitivity and specificity relative to both Hospital Anxiety and Depression Scales (HADS) and Brief Symptoms Inventory-18 (BSI-18) cut-off scores for general caseness and more severe psychological distress, respectively. HADS (Costantini et al., 1999) and BSI-18 (De Leo et al., 1993) are the most renowned instrument for the screening of psychological distress in cancer patients.

### Data Analysis

Data were anonymized and analyzed using the Statistical Package for Social Sciences for Windows, version 21.0. Dichotomous variables were examined using the chi-squared test. Analysis of variance (ANOVA) was used to analyze the differences among group means. We used multivariate regression modeling (stepwise regression) to examine predictors of distress thermometer score at admission for day-surgery.

## Results

A total of 177 patients were asked to complete the Distress Thermometer and the PL during the observational window of 1 week. Out of 177 patients, 154 patients filled in the questionnaire at admission for day surgery giving a follow-up response rate of 87%.

The mean age was 56 years (SD, 12.3; range, 19–91), and 128 (83%) were women. Half of the patients have a high school diploma (49.7%). The most common referral source was breast cancer (64 patients, 42.2%), followed by urologic surgery (16.2%), plastic surgery (14.2%), gynecologic surgery (%), and thoracic surgery (7.1%).

The majority of patients lived outside the region of the hospital, (52.6%, *N* = 81) and the great majority of patients had a previous diagnosis of cancer (72.7%, *N* = 112).

Characteristics of the patients are included in **Table 1**.

**Table 1:** Patients included.

Characteristics	*N*	(Min/Max); %
Mean Age	56	(19–91)
Gender		
Male	26	17
Female	128	83
Level of education		
Elementary school	12	8.1
Lower secondary school	31	20.8
High -school	74	49.1
University	32	21.5
Region of provenience		
Lombardy	69	46
Out of Lombardy	81	54
Type of surgery		
Senology	65	42.2
Urology	25	16.2
Plastic surgery	22	14.2
Gynecology	13	8.4
Thoracic Surgery	11	7.1
Laparoscopy	7	4.5
Head and neck surgery	5	3.2
Interventional radiology	4	2.6
Sarcoma surgery	2	1.3
Previous Cancer Diagnosis		
Yes	112	72.7
No	42	27.3

### Level of Distress

We divided the level of distress into mild (from 1 to 5), moderate (from 6 to 7) and severe (>8). While 13% of patients (*N* = 20) defined themselves as not distressed at all (score = 0), 49.4% of the sample declared a mild level of distress (*N* = 76). The rest of the patients showed a level of attention for distress: 19.5% perceived a moderate level of distress (*N* = 30) and 18.2% declared a severe level of distress (*N* = 28).

### Problems List

The PL developed by the Distress Management Guidelines Panel of the NCCN was used for the current evaluation. Specifically, the PL contained 34 problems frequently experienced by individuals with cancer, classified into the following categories: Practical Problems, Family Problems, Emotional Problems, Spiritual/Religious Concerns, Physical Problems. A total of 55% of patients presented at least three difficulties in the PL. Issues in the domain of emotional problems appeared predominant, especially nervousness, and worry, while in the domain of physical problems fatigue appeared as the most significant. Female patients showed a higher levels of distress than did male patients (*F* = 3.75; *p* = 0.005). Finally, mean differences in respect to age, prior diagnosis of tumor and region of living did not result as significant.

Distress scores correlated significantly with numbers of problems in different categories (**Table 2**). However, the strongest correlation of distress was with emotional problems and physical problems.

**Table 2:** Predictors of distress thermometer score.

Linear Regression model			Multivariate analysis		
Predictors	Regression coefficient (beta)	*P*-value	Mean squares	*F*	Significance
Emotional problems	0.655	**0.000^∗∗^**	33.242	11.113	**0.000^∗∗^**
Physical problem	0.174	**0.019^∗∗^**	13.768	2.532	**0.014^∗∗^**
Relational problems	0.068	0.396	1.235	0.227	–
Spiritual problems	0.074	0.319	1.250	0.230	–
Practical problems	0.018	0.842	20.000	3.678	–
Age	0.069	0.350	0.114	0.021	–
Gender	0.059	0.310	2.774	0.510	–
Region of living	0.020	0.810	12.335	2.268	–
Prior diagnosis of tumor	0.009	0.916	1.250	0.230	–
Emotional problems ^∗^ Physical problems			86.06	10.911	**0.002^∗∗^**
Emotional problems ^∗^ Physical problems^∗^age			66.28	9.523.	**0.005^∗∗^**

*Bold values are significant.*

### Regression Analysis

Stepwise logistic regression was used to predict the most important independent variables on distress. Independent variables included the checklist of the DT, socio-demographic variables (gender, age, region of living, and level of education), and clinical variable (previous tumor diagnosis). The strongest predictor of final DT score using multivariate regression analysis was the number of emotional problems (*P* = 0.002) followed by the number of physical problems (*P* = 0.026). About the checklist, practical problems (*P* = 0.097), and spiritual/religious concerns (*P* = 0.830) were not significant. Similarly, gender (*P* = 0.310), age (*P* = 0.068), region of living (*P* = 0.461), level of education (*P* = 0.862), prior diagnosis of tumor (*P* = 0.836) were not indicative predictors of distress levels.

An integrative multivariate analysis confirmed our results. Interestingly, we found a significant cumulative interaction effect between emotional problems and physical problems (*P* = 0.002) and age (*P* = -005). In this situation, we found a significant main effect of both emotional and physical effect and we found an interaction effect of both factors on distress. Age, as a main effect does not appear to be significant. However, age shows a cross-over interaction when it is calculated together with emotional and physical problem. Age (*per sè*) has not an overall effect on stress, but there is a crossover interaction. Mean differences shown by planned comparison have shown a significant difference between younger and older patients: older patients with higher physical and emotional problems seem to be more exposed to distress.

## Discussion

The DT was developed and validated for screening cancer patients for psychological distress, as suggested by the National Comprehensive Cancer Network in 2012. Day surgery facilities are a recent development in clinical oncology practice and have come about mainly as a result of a cost-effectiveness policy approach. There is a lack of data in the literature regarding the prevalence of psychological distress in this setting: in a large study by Herschbach et al. (2008) the distress of 6365 cancer patients was analyzed on the basis of an expert rating with a specific focus on the relevance of setting variables in causing distress (type of hospital, type of treatment) and data from day surgery settings were not available. Thus, the current study was aimed at filling in this missing data, measuring the rates of distress in a sample of patients being hospitalized in a multidisciplinary day surgery facility of a comprehensive cancer center.

From the results of this explorative study one may propose that the DT accompanied by the PL may offer a useful screening instrument with which to improve the management of cost-effective day surgery activities, promoting a multidisciplinary practice which, in the light of the lack of data in this area will include psycho-social aspects.

These findings suggest that distress evaluation and management appears to be challenged in a critical care setting such the Day Surgery. Such a setting is characterized by care delivery by multiple providers, and patients being affected by multiple and different conditions, where time, severity, and urgency have always to be well managed. In this light, the results of this pilot study may give valid explorative information for clinical decision-making practice.

Patients showed a clinically significant distress prior to their intervention and this information is still underestimated and rarely evaluated in routine day surgery activities (Marla and Stallard, 2009), even though it became mandatory for Cancer Center accreditation as prescribed by the American College of Surgeons Commission on Cancer (Zebrack et al., 2015). The standard requires that cancer-treating institutions develop and implement a process to integrate and monitor on-site psychosocial distress screening and referral for the provision of psychosocial care.

In our study, distress was not related to age, education, type of cancer, or a diagnosis of tumor. This confirms data from the literature on Italian patients (Grassi et al., 2013). However, a cumulative interaction effect was found between age and physical problems.

It is also interesting to note that distress was not related to place of living. The latter is an important finding, because no other study has sought to examine the possible role played by place of living in influencing scores on the DT in patients entering a day surgery division in which transfers from outside the region for a brief hospital stay could represent an additional source of distress.

Our study showed that distress was greater in women than in men confirming data reported in the literature on Italian cancer population (Grassi et al., 2011).

DT levels were significantly correlated with emotional and practical problems. This is a significant result that highlights the importance of psychological components in psycho-social oncology even in those care settings characterized by the short length of hospital stay: resources should be placed to properly address the emotional needs of cancer patients in a 1-day setting for example by means of a telephone-based psychological referral, an idea which has been demonstrated to be effective in cancer settings (Arnaboldi et al., 2010).

Since a process of evaluation and appropriate referral should be considered as the central part of a screening program (Pirl et al., 2014) our main purpose for the future is to properly address significant levels of distress via developing structured psychological interventions compatible with a day surgery environment especially to address elderly patients’ needs since they seem to be frailer and more demanding from a psycho-social point of view.

Another important future aim is to help patients to become more empowered and able to cope with stress. As several studies both in the oncological and non-oncological settings have shown (e.g., Schulz et al., 2013; Riva et al., 2014), “effective coping skills allow patients to retain as much of their lives in spite of a medical condition that is chronic or difficult to diagnose or treat” (Schulz et al., 2013; Riva et al., 2015).

Successful implementation of distress screening has been demonstrated to be related to the training, preparation and coordination of healthcare providers and staff in the application of the screening protocol (Lucchiari and Pravettoni, 2012; McLeod et al., 2014). Thus, another main purpose for the future is that of developing an education program to support screening for distress which is feasible in a day surgery setting.

These results are in accordance with the data showing that the presence of emotional and practical symptoms can increase the perception of distress (Grassi et al., 2004; Arnaboldi et al., 2010; Schreiber and Brockopp, 2012).

In the future it will be important to investigate the presence of any correlation between patient psychological distress at admission for day surgery and patient distress at follow up.

Regarding the intelligibility and clarity of the DT, the complete response rate was high. We would nonetheless highlight the importance of healthcare workers ensuring that the patient understands how to fill in the DT questionnaire. Indeed, we did not analyze the 13% of the cases because they were incomplete.

The present study is not without certain limitations. First of all, the sample was represented by a non-probability sampling characterized by people who underwent surgery, consecutively.

Second, the cancer site was mostly the breast; this did not permit us to comprehend, in a more exhaustive way, the possible differences in DT scores using many cancer sites.

Despite this limitation, this study, to the best of our knowledge, reports DT findings in one of the first cohorts of day surgery populations.

## Ethics Statement

This study was approved by Istituto Europeo di Oncologia Ethic Committee. Each participant receive an Information Sheet and an Informed Consent by GM or PA (authors of the manuscript). Each participant signed an Informed Consent.

## Author Contributions

PA conceived the research, collected data and wrote the manuscript. SR conceived the research, and wrote the manuscript. VV, LT, and GM collected data. GP supervised the research and wrote the manuscript. All the authors agreed on the final version of this manuscript.

## Conflict of Interest Statement

The authors declare that the research was conducted in the absence of any commercial or financial relationships that could be construed as a potential conflict of interest.
